# Cell-Free, Embryo-Specific sncRNA as a Molecular Biological Bridge between Patient Fertility and IVF Efficiency

**DOI:** 10.3390/ijms20122912

**Published:** 2019-06-14

**Authors:** Angelika V. Timofeeva, Vitaliy V. Chagovets, Yulia S. Drapkina, Nataliya P. Makarova, Elena A. Kalinina, Gennady T. Sukhikh

**Affiliations:** Kulakov National Medical Research Center of Obstetrics, Gynecology, and Perinatology, Ministry of Health of Russia, Ac. Oparina 4, Moscow 117997, Russia; vvchagovets@gmail.com (V.V.C.); julia.drapkina@gmail.com (Y.S.D.); npmakarova@gmail.com (N.P.M.); e_kalinina@oparina4.ru (E.A.K.); gtsukhikh@mail.ru (G.T.S.)

**Keywords:** miRNA, piRNA, NGS, RT-PCR, embryo culture medium

## Abstract

Small noncoding RNAs (sncRNAs) are key regulators of the majority of human reproduction events. Understanding their function in the context of gametogenesis and embryogenesis will allow insight into the possible causes of in vitro fertilization (IVF) implantation failure. The aim of this study was to analyze the sncRNA expression profile of the spent culture media on day 4 after fertilization and to reveal a relationship with the morphofunctional characteristics of gametes and resultant embryos, in particular, with the embryo development and implantation potential. Thereto, cell-free, embryo-specific sncRNAs were identified by next generation sequencing (NGS) and quantified by reverse transcription coupled with polymerase chain reaction (RT-PCR) in real-time. Significant differences in the expression level of let-7b-5p, let-7i-5p, piR020401, piR16735, piR19675, piR20326, and piR17716 were revealed between embryo groups of various morphological gradings. Statistically significant correlations were found between the expression profiles of piR16735 and piR020401 with the oocyte-cumulus complex number, let-7b-5p and piR020401 with metaphase II oocyte and two pronuclei embryo numbers, let-7i-5p and piR20497 with the spermatozoid count per milliliter of ejaculate, piR19675 with the percentage of linearly motile spermatozoids, let-7b-5p with the embryo development grade, and let-7i-5p with embryo implantation. According to partial least squares discriminant analysis (PLS-DA), the expression levels of let-7i-5p (Variable Importance in Projection score (VIP) = 1.6262), piR020401 (VIP = 1.45281), and piR20497 (VIP = 1.42765) have the strongest influences on the implantation outcome.

## 1. Introduction

Infertility affects almost 15% of couples globally. One of the most effective methods to treat infertility is assisted reproductive technology (ART), which covers a wide spectrum of treatments. Although ART has the highest pregnancy and live birth rates, it still has limited efficacy. Only 32.3% of ART cycles performed for the first time in women under 37 years old result in the birth of a healthy child. Patients aged more than 37 years old who commence in vitro fertilization (IVF) treatment have only a 12.3% success rate. A successful result in ART programs depends on a balanced interaction of a top-quality embryo with an optimally receptive endometrium.

With the worldwide move towards single embryo transfer, there is a renewed focus on developing a reliable method of assessment of embryo viability. The ability to select an embryo with the highest developmental and implantation potential is paramount to maximizing the probability of attaining a successful pregnancy.

Numerous systems have been devised to grade and rank embryos [1]. One of the most common grading systems is based on morphological criteria developed by Gardner et al. [2]. The grading usually considers the morphology of an inner cell mass (ICM) and trophectoderm (TE) for embryos at the blastocyst stage as well as the degree of expansion of the blastocyst cavity. However, sometimes, it is difficult to interpret data on morphological characteristics and morphological traits as they do not fully reflect the physiology of the embryo and its chromosomal complement. Moreover, even good quality embryos do not always implant.

Different biomarkers of embryo quality and implantation potential have been proposed in addition to morphological criteria. They include noninvasive metabolomic and proteomic profiling of embryo culture media [3,4,5]. However, these biomarkers have their own disadvantages. The metabolomic profile of embryo culture media requires the use of very sensitive instruments which can detect even slight changes in media composition. This is crucial because embryo metabolomic activity depends on the media. Embryo proteomic profile assessment remains inaccurate, because one must enrich media with exogenous major proteins to assure normal embryo development. 

Cells and tissues that are involved in human reproduction processes are unique because they constantly undergo dramatic reorganization during gametogenesis and embryogenesis at both transcriptomic and proteomic levels. Recently, scientists have started to focus on cell proteomic profile changes which are controlled by non-coding RNAs. MicroRNAs (miRNAs), small endogenous interfering RNAs (siRNAs), and piwi-interacting RNAs (piRNAs) have the most crucial roles among these molecules [6]. Most miRNAs are transcribed by RNA polymerase II [7]. The result of this process is the formation of a primary transcript (pri-miRNAs) which is 2000–4000 nucleotides in length, has a 5’-7-methyl-guanosine cap structure, a 3’-poly (A) tail, and a complex secondary structure. Further processing of pri-miRNAs continues in the cell nucleus under the control of conserved RNase III Drosha and the DiGeorge Syndrome Critical Region Gene 8 (DGCR8) cofactor with the formation of a hairpin structure, 70–100 nucleotides in length, termed precursor miRNA (pre-miRNA). The resulting pre-miRNA is transported to the cytoplasm via a process that involves Exportin-5 and RAN (RAs-related Nuclear protein) GTPase. The pre-miRNA is further cleaved by RNase III Dicer and Tar RNA-binding protein (TRBP) to generate a short, partially double-stranded (ds) RNA with approximately 22 nucleotides in which one strand is the mature miRNA. Afterwards, the mature miRNA strand is taken up by a multi-protein complex that is identical to the RNA Induced Silencing Complex (RISC) that supports RNA interference (RNAi) and miRNA-bound complex functions to regulate translation. Meanwhile, another strand of miRNA undergoes degradation. 

The biogenesis of piRNAs is less studied in comparison with that of miRNAs and siRNAs. It does not require Dicer, because the precursor piRNAs are single-stranded [8]. There are several classes of piRNAs according to next generation sequencing (NGS) data [9]. The class I piRNAs are produced by clustered genetic loci. The piwi-protein-mediated cleavage of the transposon RNA target produces the class II piRNAs. The last group of piRNAs originates from different genomic regions, including three prime untranslated regions of some mRNAs. This fact demonstrates that piRNAs might regulate the level of gene expression in addition to chromosomal rearrangement maintenance by repressing transposable element expression. The piRNA amplification model and transposons neutralization occur via the ping-pong mechanism in germ cells [10].

miRNAs are synthesized in the granulosa and cumulus cells and secreted into the follicular fluid. The influence of these molecules on oocyte maturation and embryo quality has been already shown [11,12,13]. The effect of female age on the miRNA profile of an oocyte has also been clearly demonstrated [14]. Moreover, miRNA expression levels depend on the degree of oocyte maturation [15]. Elke F. Roovers et al. showed dramatic expression of piRNAs in human, bovine, and macaque oocytes [16]. The piRNA profile in oocytes was similar to that in sperm at the pachytene stage. The principal role of this stage during meiosis is the regulation of transposons expression and, consequently, it controls genome instability [16]. The role of piRNAs and miRNAs during spermatogenesis is described in detail in a review article [17]. This article focuses on the epigenetic regulatory functions of DNA modification, mRNA translation and stability, and the maintenance and self-renewal of germ stem cells. The crucial role of sperm small non-coding RNAs (sncRNAs) produced after fertilization in embryogenesis, implantation, and subsequent embryo development has been recently proven [18]. It has also been shown that successful embryonic development immediately after fertilization depends on coordinated maternal mRNA degradation and zygotic genome activation, resulting in embryonic mRNA production and protein translation. It has been found that piRNAs, miRNAs, and siRNAs are essential for the maternal–zygotic transition [19,20,21].

In the light of the above, the development of non-invasive methods for predicting the onset of pregnancy and its further development is a highly topical problem at present. An example of such a method is the evaluation of miRNAs specific for the villus and decidual tissues in the peripheral blood plasma of women in both the first trimester of pregnancy and the day of embryo transfer (ET) in ART programs [22]. It was demonstrated that MiR (miR-23a-3p, 27a-3p, 29a-3p, 100-5p, 127-3p, and 486-5p) expression levels significantly changed in the group of women with recurrent miscarriage compared to the group with normal pregnancy, but the clinical pregnancy outcome could not be predicted in the IVF/ET (in vitro fertilization/embryo transfer) patients. Another diagnostic method for predicting pregnancy is to evaluate the expression profile of sncRNAs secreted by embryo cells into their culture media. To the best of our knowledge, very few studies have considered the interactions that exist between sncRNA expression levels in culture medium and embryo implantation potential [23,24,25]. To create more effective treatment for infertile couples in the IVF program, the implication of sncRNAs from spent culture medium in the formation of the morphofunctional characteristics of gametes and resultant embryos, as well as in embryo development and implantation potential, should be investigated. Therefore, the present study was aimed at revealing and analyzing such interactions.

## 2. Results and Discussion

### 2.1. Identification of Embryo-Specific sncRNAs

**Figure 1:**
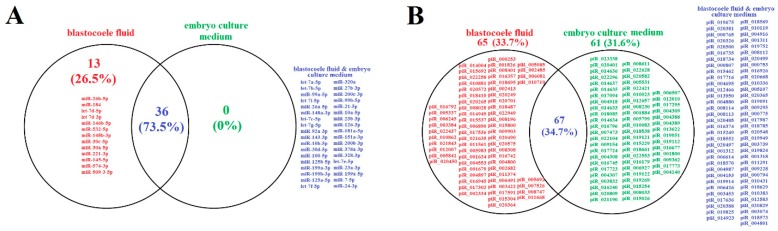
Venn diagrams for microRNA (miRNA) expression pattern (**A**) and piwi-interacting RNA (piRNA) expression pattern (**B**). The sncRNAs (miRNA and piRNA) unique to the blastocoele fluid are written in red; sncRNAs written in green are unique to the blastocyst spent culture medium by the fifth day after fertilization; sncRNAs are written in blue provided that they are detected both in the blastocoele fluid and in the blastocyst spent culture medium.

Deep sequencing was performed to identify embryo-specific sncRNAs. A blastocyst of excellent quality (4AA) from one couple was enrolled in the study. The fraction of RNA secreted both into the blastocoele and into its culture medium on day 5 after fertilization was analyzed. Sequence reads aligned to miRBase v21 and piRNABase with a count of at least 10 were compared by plotting Venn diagrams of the miRNA expression pattern (Figure 1A) and piRNA expression pattern (Figure 1B). Figure 1 demonstrates a wider spectrum of piRNAs (132 and 128 subtypes in blastocoele liquid and embryo culture medium, respectively, Figure 1B) in comparison with miRNAs (49 and 36 subtypes in blastocoele liquid and embryo culture medium, respectively, Figure 1A). Among them, 73.5% miRNAs and 34.7% piRNAs are secreted by the embryo into both the blastocoele liquid and the embryo culture medium. All of the miRNAs secreted by the embryo into the culture medium were detected in the blastocoele liquid. As for the piRNAs (Figure 1B), 33.7% were uniquely expressed in the blastocoele liquid and 31.6% in the embryo culture medium. 

### 2.2. Real Time PCR Analysis of sncRNA Revealed by NGS

miRNAs and piRNAs characterized by detectable PCR signals with Ct < 35 cycles (Table 1) and specific PCR products (a single peak of the amplification product melting curve) were selected for subsequent analysis by RT-PCR in 87 RNA samples from embryo culture medium on day 4 after fertilization.

**Figure 2:**
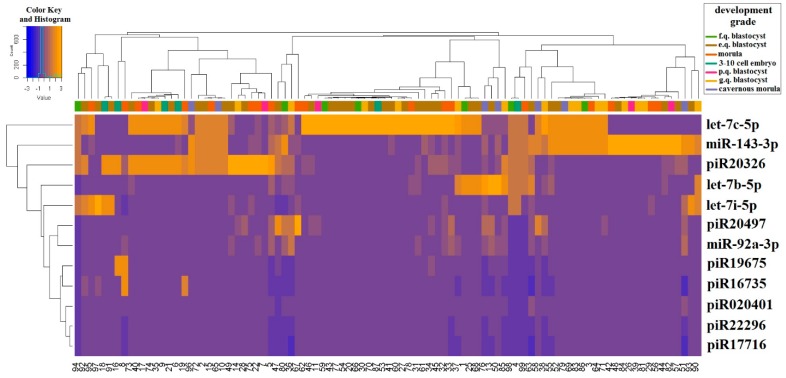
Two-dimensional hierarchical clustering of real-time RT-PCR data on sncRNAs from 87 embryo cultivation medium samples. E.q.—excellent quality, g.q.—good quality, f.q.—fair quality, p.q.—poor quality.

**Figure 3:**
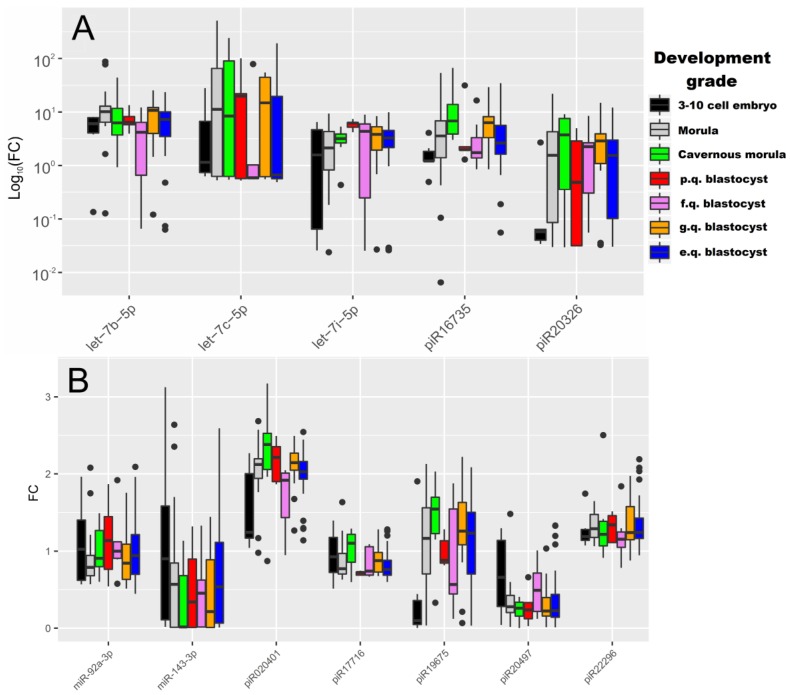
sncRNA expression level fold change in the culture medium of embryos of different development grade. (**A**) sncRNA expression level fold change (FC) with values varying from 0.01 to 400 (data are presented on a logarithmic scale). (**B**) sncRNA expression level FC with values varying from 0.1 to 3.5 (data are presented on a linear scale). E.q.—excellent quality, g.q.—good quality, f.q.—fair quality, p.q.—poor quality.

Two-dimensional hierarchical clustering of real-time RT-PCR data was performed to classify 87 embryo cultivation medium samples according to the degree of expression profile similarity of the studied sncRNAs. The Pearson and Euclidean distance correlations were used to calculate the difference between dendrogram nodes. The heat map for the RT-PCR data clustering is presented in Figure 2. The sncRNA clustering patterns did not clearly separate culture media samples from excellent, good, fair, and poor-quality embryos according to Gardner grading scale, or from morulas, cavernous morulas, and 3–10 cell embryos. This may be explained by undulating changes in sncRNA expression which depend on the embryo development stage. Certain patterns of the studied sncRNAs expression throughout the development stages and inside subgroups of blastocysts of different quality can be observed in the boxplot in Figure 3. For example, the trends of the piR19675 and piR020401 expression level medians are similar for the “morula”, “good quality blastocyst”, and “excellent quality blastocyst” groups but are dramatically different in other groups. Meanwhile, the median trend in the let-7b-5p expression level is similar in the “3–10 cell embryo”, “cavernous morula”, and “poor quality blastocyst” groups. The level of let-7b-5p expression increases in “morula” and “good and excellent quality blastocyst” groups and decreases notably in the “fair quality blastocyst” group. The Mann–Whitney U-test revealed statistically significant differences in the expression levels of let-7b-5p, let-7i-5p, piR020401, piR16735, piR19675, and piR20326 in the culture medium from embryos of various development grades (Table 2). Moreover, blastocysts of different quality according to Gardner’s grading scale were shown to differ significantly from each other in expression levels of let-7i-5p, piR020401, and piR17716. The obtained data are in good agreement with those of Abd El Naby et al., who demonstrated miRNA expression dynamics during the preimplantation stage period from bovine zygote to blastocyst [15].

**Figure 4:**
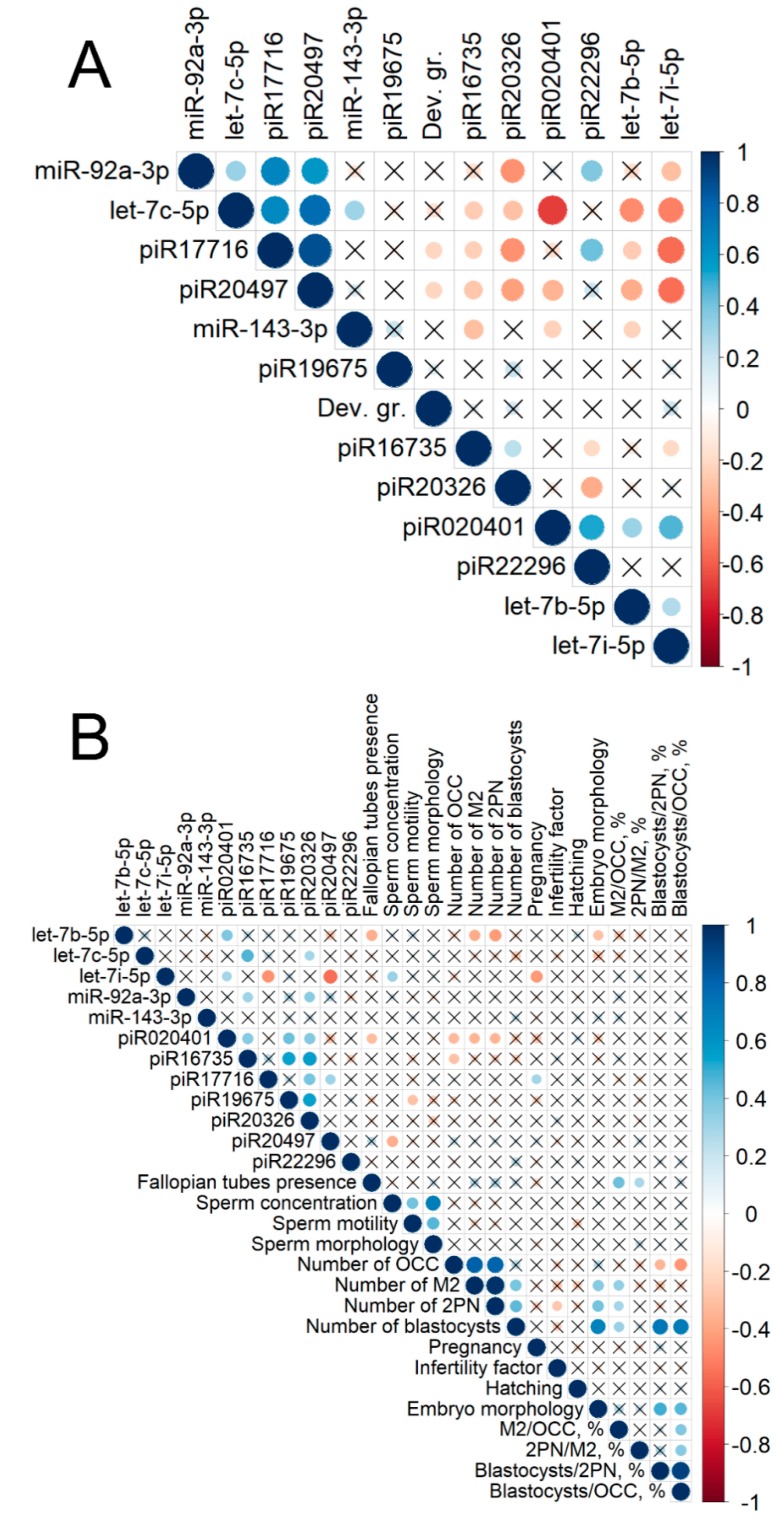
Correlation matrix based on the non-parametric Spearman rank correlation method. Significant (*p* < 0.05) correlations are indicated by a dot, non-significant correlations are indicated by a cross, positive correlations are marked in blue, and negative correlations in red—the more significant the correlation, the larger the dot size. (**A**) Correlation analysis of the sncRNA expression level in 87 embryo cultivation medium samples and the quality of embryos. (**B**) Correlation analysis of the sncRNA expression level in 48 cultivation medium samples of embryos transferred to the uterus and the following parameters: fallopian tube presence—the presence of fallopian tubes in the female of each couple; sperm concentration—spermatozoid count per milliliter of ejaculate from the male of each couple; sperm motility—percentage of linearly motile spermatozoids from the male of each couple; sperm morphology—percentage of morphologically normal spermatozoids from the male of each couple; number of OCC—the number of oocyte–cumulus complexes from the female of each couple; number of M2—metaphase II oocyte number from the female of each couple; number of 2PN—two pronuclei embryo numbers from each couple; number of blastocysts—blastocyst number of each couple; pregnancy—the development of pregnancy after embryo transfer into the uterus; infertility factor—primary or secondary infertility in the female of each couple; hatching—embryo hatching before transfer to the uterine cavity; dev. gr.—embryo development stage and quality according to the Gardner grading scale; embryo morphology—morphological parameters of embryos according to the Gardner grading scale; M2/OCC, %—metaphase II oocyte number as a percentage of OCC; 2PN/M2, %—number of two pronuclei embryos as a percentage of M2 oocyte; blastocysts/2PN, %—blastocyst number as a percentage of 2PN embryos; blastocysts/OCC, %—blastocyst number as a percentage of OCC.

Since embryo sncRNA expression patterns which contribute to the embryo quality and development potential depend on the degree of oocyte maturation, concomitant diseases of the female reproductive and endocrine systems, and also, sperm characteristics, a correlation matrix was calculated and plotted to reveal these interactions (Figure 4). The analyzed samples ranged, according to the embryo development grading scale, in the following way: “3–10 cell embryo” < “morula” < “cavernous morula” < “poor quality blastocyst” < “fair quality blastocyst” < “good quality blastocyst” < “excellent quality blastocyst”. Considering all 87 samples (Figure 4A), a reliable correlation of sncRNA expression profiles was found both within one class and between classes of these molecules. The expression levels of piR17716 and piR20497 negatively correlated with the embryo development grade, positively correlated with each other and with miR-92a-3p and let-7c-5p, and negatively correlated with piR16735, piR20326, let-7b-5p, and let-7i-5p. In contrast, the expression level of let-7c-5p negatively correlated with the expression of other Let7 family members (let-7b-5p and let-7i-5p) and piR020401. Such a complex interrelation of sncRNA expression patterns in the culture media of embryos of various development grades is probably a manifestation of the fine system of signaling pathway regulation necessary for the implementation of the embryogenesis program. The crucial role of miRNAs and piRNAs in the post-transcriptional gene regulation is already well known [12,13,14,15,16,17,18,19,20,21].

A correlation matrix with 48 samples of culture media from embryos which were transferred to the uterine cavity was calculated (Figure 4B) to analyze the interaction between pregnancy, the quality of oocytes retrieved, sperm quality, the number of blastocysts obtained, and the expression profile of sncRNAs. The correlation matrix revealed that the expression level of the Let7 family in embryo culture media can be a potential biomarker for IVF efficiency prognosis. For example, the expression level of let-7b-5p negatively correlated with both the embryo development grade and the number of M2 oocytes and 2PN cells. Moreover, the M2 oocyte number as a percentage of oocyte–cumulus complexes (OCC) and the 2PN embryos number as a percentage of the M2 oocyte number had positive correlations with fallopian tube presence. Many experts have assumed the effects of an epithelium in the fallopian tubes on oocyte maturation and the ability to fertilize [26]. In turn, the let-7b-5p expression level was positively correlated with the piR020401 expression profile, which had a negative correlation with the number of OCC, M2-oocytes, and 2PN-cells. In addition, this piRNA was also positively correlated with the expression patterns of piR16735, piR19675, and piR20326. As for the piR16735 and piR1967 expression patterns, the former was negatively correlated with the OCC number, and the latter was negatively correlated with sperm motility. Meanwhile, the sperm concentration was positively correlated with sperm progressive motility and normal sperm morphology.

A promising interaction between let-7i-5p, piR17716, and piR20497 was found. The expression pattern of let-7i-5p had a negative correlation with the piR17716 and piR20497 expression profiles. Along with this, the piR17716 expression pattern was positively correlated with implantation, while the piR20497 expression profile had a negative relationship with sperm concentration. However, for let-7i-5p, an inverse correlation was obtained. A negative correlation of its expression with the implantation rate and a positive correlation with the sperm concentration were found.

As for let-7c-5p, a positive correlation of its expression with piR16735 and piR19675 was observed. piR16735 expression was negatively correlated with OCC, whereas piR19675 expression had a negative relationship with the number of motile sperm. It was difficult to identify whether the analyzed sncRNA in the embryo culture medium originated from gametes involved in fertilization or from the activated embryo genome after the maternal–zygotic transition, but there is strong evidence for the contribution of sperm RNA to the embryo developmental potential and its implantation ability according to the literature data [18].

The number of OCCs collected in one stimulated cycle correlated negatively with the number of blastocysts as a percentage of OCCs or 2PN (Figure 4B). The obtained results were consistent with literature data, indicating that the number of retrieved oocytes in a stimulated cycle has a negative relationship with the number of blastocysts obtained [27,28].

The relationship of the individual ovarian response to gonadotropins in IVF programs and post-transcriptional regulation of genes responsible for oocyte maturation was shown by Cengiz Karakaya et al. [11]. They found that a poor ovarian response to IVF is associated with up-regulation of 16 miRNAs and down-regulation of 88 miRNAs in cumulus cells. It is possible that the spectrum of sncRNAs secreted by a fertilized oocyte after four days of in vitro cultivation and correlated with the number of OCC and M2-oocytes may be a consequence of the post-transcriptional regulation of genes through cumulus–oocyte communication. 

**Figure 5:**
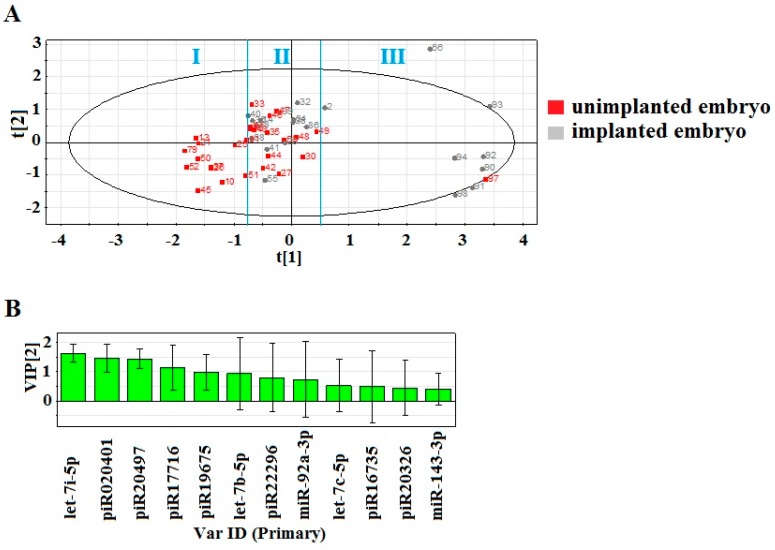
Partial least squares discriminant analysis (PLS-DA) of 2^−^^ΔΔCt^ RT-PCR data on the expression of sncRNAs in the 48-embryo culture medium samples. Arabic numerals denote the sample number. Roman numerals (I, II, III) denote the cluster of samples depending on the result of embryo transfer into the uterine cavity and the profile of RNA expression in the cultivation medium. (**A**) score plot with the imposition of information of the sncRNA expression level on the results of embryo transfer into the uterine cavity, (**B**) Variable Importance in Projection (VIP) score.

The PLS-DA model based on RT-PCR data was developed to study differences between the culture medium from embryos which implanted or did not implant. The fold change of the sncRNA expression level in each of the 48 samples was used for the model (Figure 5). Figure 5A represents the score plot of the developed PLS-DA model. Three clusters of data points can be distinguished. The first one has an abscise of less than −0.75 and represents the embryos which failed to implant despite there being an appropriate quality. The second cluster lies between −0.75 and 0.5 of the abscise and contains data points corresponding to embryos with similar morphological properties (excellent, good, and fair quality according to Gardner’s grading scale) and molecular biomarkers (sncRNA expression profile), but some of them implanted (highlighted in gray), and some of them failed to implant (highlighted in red). This phenomenon can be explained by independent factors irrelevant to the embryo quality, e.g., endometrial receptivity, chronic inflammation, or this might have happened because of the other sncRNAs which were not analyzed in the current study. The third cluster is characterized by an abscise greater than 0.5. This cluster corresponds to embryos with high implantation potential, according to morphological grading scale and molecular biomarkers, that successfully implanted. The contribution of sncRNA to the distribution of the data points on the score plot can be estimated by the Variable Importance in Projection (VIP) score (Figure 5B). The following sncRNA have VIP > 1 and those with the highest impact are let-7i-5p (VIP = 1.6262), piR020401 (VIP = 1.45281), piR20497 (VIP = 1.42765), and piR17716 (VIP = 1.14438). Notably, the expression profile of these sncRNAs has a strong correlation with sperm quality, the number of OCCs, oocyte maturity, and oocyte fertilization ability (Figure 4B). Thus, one can suppose that these molecules contribute to the embryo implantation potential. In addition, the Mann–Whitney test revealed significant differences in let-7i-5p and piR020401 expression levels in the culture media from embryos of clusters II and III which succeeded in implanting (highlighted in gray in Figure 5A) in comparison to embryos from cluster I which failed to implant (highlighted in red in Figure 5A): 2.38 ± 2.25 vs. 5.20 ± 1.84, *p* < 0.001 for let-7i-5p; 1.85 ± 0.46 vs. 2.28 ± 0.35, p = 0.005 for piR020401. The culture media from the embryos with implantation failure (highlighted in red in Figure 5A) differed significantly in let-7i-5p expression when comparing the embryos of cluster I and cluster II: 5.20 ± 1.84 vs. 3.41 ± 1.79, p = 0.018. Consequently, let-7i-5p can be proposed as a marker of the embryo’s implantation potential on the 4th day after fertilization. 

Thus, the PLS-DA model developed by us reflects the implantation potential of an embryo according to the expression profile of sncRNAs in the culture medium on the 4th day after fertilization but cannot be used to predict the onset of pregnancy and its development. The fact remains that the embryo transferred into the uterine cavity, and endometrial cells are able to reciprocally exchange signals, in particular, in the form of secreted extracellular vesicles containing small non-coding RNA. This topic is discussed in detail in a review article by Ferlita A. et al. [29]. Even if the molecular-biological profile of the embryo is normal and the spectrum of secreted sncRNAs corresponds to an embryo with high implantation potential, abnormal secretion of RNA molecules and proteins by the endometrium can adversely affect the embryo implantation process. Thus, to improve the performance of IVF programs before embryo transfer to the uterus, it is necessary not only to evaluate the expression profile of small non-coding embryonic RNA, as proposed in this work, but also to assess endometrial receptivity, in particular, focusing on the extracellular sncRNAs present in endometrial fluid during the window of implantation.

### 2.3. Identification of piRNA Targets

piRNA sequences were annotated using the piRBase database (Available online: http://www.regulatoryrna.org), which contains information on the piRNA genome location (hg38) (Table 3). Studied piRNAs are mapped to the transposable elements (TE) such as long interspersed elements (LINEs) and short interspersed elements (SINEs), including Alu elements. These data are in good agreement with published information confirming that most mammalian genomes are dominated by LINE and SINE retrotransposons [30], where LINE and SINE are the most abundantly represented TE classes in the bovine testis, oocyte, and zygote pilRNAs, representing over 50% of all TE mapped reads [31]. Some piRNAs, analyzed in the current study, were mapped to tRNA (tRNA-GluGAG, tRNA-AlaGCY, tRNA-Gly-GGG, tRNA-GlyGGY, tRNA-GlyGCC and tRNAValAAC) and rRNA species, which are specifically processed into piRNAs. Recent studies have also revealed that some tRNA-derived small RNAs associate with the Argonaute (AGO) proteins or P-element induced wimpy testis proteins (PIWI) [32], and the accumulation of small tRNAs and rRNAs and their association with the RNA interference machinery seems to be characteristic of highly proliferative cells and tightly controlled to avoid apoptosis [33]. Additionally, we found out from piRBase that piRNAs are mapped not only to the repeat elements but also to the region of protein coding genes, and these piRNAs are referred to as gene-derived, in particular, *LY6G5C*-, *EFCAB11*-, *VAC14*-, *COLQ*-, and *PTGES3L*-derived piRNAs (Table 3). These findings are in accordance with the published literature and confirm that some mRNAs in flies and vertebrates are known to be processed into piRNAs [21,34,35,36].

Recent studies have suggested that piRNAs have the potential to target mRNAs in addition to their traditional transposon-derived transcripts [37,38,39]. piRNA target data have been collected from literature in piRBase and related to mice and fruitflies but not to human piRNA until now. Therefore, BLAST (Available online: https://blast.ncbi.nlm.nih.gov) was used to identify human piRNA overlap with protein coding genes. mRNA was considered a potential target for piRNA if the mapping direction for the piRNA–mRNA pair was opposite. This approach was proposed by S. Russell for Bowtie application [31]. Lists of potential gene-targets were obtained for hsa-piR020401, hsa-piR019675, and hsa-piR022296 (Table 3). We focused on the 25 gene targets of hsa-piR020401 and hsa-piR019675. The expression profiles of these molecules correlated with the OCC, M2, 2PN number, and the percentage of sperm with linear motility. A Gene Ontology (GO) analysis of these genes in the PANTHER Classification System (Available online: http://pantherdb.org/) showed that within the molecular function category, 28% of genes are related to binding processes (including transcription factor like 5 (*TCFL5*), Rho Guanine Nucleotide Exchange Factor 10 Like (*ARHGEF10L*), p21-activated kinase 3 (*PAK3*), Eukaryotic Translation Elongation Factor 1 Delta (*EEF1D*), Polyhomeotic-like protein 3 (*PHC3*), RNA Binding Motif Protein 28 (*RBM28*), Feline Encephalitis Virus-Related Kinase (*FER*)), 24% of genes are involved in catalytic activity (Methyltransferase Like 22 (*METTL22*), DNA polymerase eta (*POLH*), *PAK3*, member of RAS oncogene family like 2B (*RABL2B*), Lipoic acid synthetase (*LIAS*), *FER*), *ARHGEF10L* is considered a molecular function regulator, Zinc fingers and homeoboxes protein 3 (*ZHX3*) and *PHC3* are implicated in transcription regulator activity, Eukaryotic Translation Elongation Factor 1 Delta (*EEF1D*) is implicated in translation regulator activity, and Calcium Voltage-Gated Channel Auxiliary Subunit Alpha2delta 1 (*CACNA2D1*) is involved in transporter activity. Therefore, it seemed important to focus on several genes whose function may be associated with reproduction. For instance, Lawrence M. Roth et al. revealed the role of *TCFL5* in normal human spermatogenesis [40]. *ARHGEF10L* is involved in the positive regulation of cytoskeleton organization, and thereby is implicated in microtubule dynamics, signal transduction, gene expression, and enzymatic regulation [41]. *PHC3* is a ubiquitously expressed member of the polycomb gene family, encoding a diverse set of regulatory proteins that are involved in the maintenance of the expression patterns that control development [42,43] and are responsible for long-term silencing of genes by altering chromatin structure through the deacetylation of histone tails and by inhibiting adenosine triphosphate (ATP)-dependent chromatin remodeling [44,45]. The loss of *PHC3* may favor tumorigenesis by potentially disrupting the ability of cells to remain in G0 [46]. *POLH* is associated with the replication of damaged DNA by synthesis across a lesion in the template strand, which allows DNA synthesis to continue beyond the lesion. Observations of Ohkumo T. et al. in the Caenorhabditis elegans suggest that *POLH* contributes to damage tolerance against UV irradiation to ensure the successful completion of embryogenesis; this provides important insights into its role in DNA damage tolerance in germ and embryonic cells [47]. *ZHX3* belong to the homeodomain transcription factor family, which is crucial for the development from embryogenesis to cell differentiation, including neuronal differentiation [48].

### 2.4. Functional Annotation of miRNA Target Genes

Potential and experimentally verified target mRNAs were determined using four separate webtools to explore the biological significance of the studied miRNAs. These webtools were DianaTools microT-CDS, DianaTools_TargetScan, DianaTools_Tarbase, and miRtargetlink. DianaTools microT-CDS and DianaTools_TargetScan were used to predict the target genes, whereas DianaTools_Tarbase and miRtargetlink allowed identification of the experimentally validated targets. Kyoto Encyclopedia of Genes and Genomes (KEGG) pathways with *p*-values of < 0.05 were united with DianaTools (Table 4) to identify signaling pathways regulated by several miRNAs at the same time. It is important to focus attention on the regulatory effects of miR-92a-3p, let-7b-5p, let-7c-5p, and let-7i-5p on signaling pathways. Their effector molecules might be involved in gameto- and embryogenesis. It is essential to mention signaling pathways regulating the pluripotency of stem cells, the extracellular matrix (ECM)–receptor interaction; adherens junctions; RNA transport; protein processing in the endoplasmic reticulum; protein digestion and absorption; ubiquitin mediated proteolysis; the Phosphoinositide 3-kinase (PI3K)-Akt serine/threonine kinase signaling pathway; the cell cycle; the Wingless-type Mouse mammary tumor virus integration site family member (Wnt)-signaling pathway; the Hippo signaling pathway; the FoxO signaling pathway; the mitogen-activated protein kinase (MAPK) signaling pathway; the transforming growth factor beta (TGFβ) signaling pathway; the p53 signaling pathway; the estrogen signaling pathway; oocyte meiosis; and valine, leucine, and isoleucine biosynthesis or degradation. For instance, the PI3K-Akt, MAPK, Hippo, and Wnt signaling pathways participate in protein synthesis, cell survival, migration, invasion, cell cycle progression, and cellular proliferation and differentiation [49,50]. The first cell differentiation event in embryogenesis occurs when the outer blastomeres of the embryo form a trophectoderm, and the remaining blastomeres form the inner cell mass (ICM), giving rise to embryonic stem cells which have the potential to self-renew and differentiate into different cell types and tissues. The balance between differentiation and self-renewal in embryonic stem cells is maintained, among others, by the Hippo pathway [50,51]. Moreover, it has been reported that the Hippo pathway can interact with other pathways to promote and maintain pluripotency. For example, the transcriptional co-activator with PDZ-binding motif (TAZ), the major mediator of the Hippo pathway, associates with Smad2/3 (directly phosphorylated by TGFβ receptors) and maintains the nuclear accumulation of Smad complexes, thereby promoting the expression of pluripotency markers (Oct4, Nanog) in response to TGFβ stimulation [51]. In turn, the TGFβ signaling pathway is modulated by deubiquitinating enzymes by regulating TGFBR1, TGFBR2, R-SMADs, co-SMAD, and I-SMAD [52]. The most crucial role in embryological events has canonical Wnt signaling, which is implicated in cell fate decisions, stem cell maintenance, body-axis determination in vertebrate embryos, and gastrulation [53]. 

In our study, statistically significant correlations were found between the expression profile of let-7b-5p and the metaphase II oocyte number, the two pronuclei embryos number, and the embryo development grade, while the expression profile of let-7i-5p correlated with the sperm count per milliliter of ejaculate and with embryo implantation. let-7b-5p and let-7i-5p regulate the FoxO signaling pathway, controlling the expression of 35 genes (Table 4), among which *FOXO1* plays the most important role. Kuscu N et al. showed that mouse FoxO1, FoxO3, and FoxO4 proteins are regulated by the PI3K/Akt signaling pathway and differentially expressed in prophase I, metaphase I, and metaphase II oocytes, as well as in fertilized oocytes, 2-cell embryos, 4-cell embryos, 8-cell embryos, morula, and blastocysts [54]. Therefore, they are implicated in oocyte maturation and preimplantation embryo development. 

According to Targetscan and Tarbase, the let-7 family regulates valine, leucine, and isoleucine biosynthesis or degradation (Table 4). Perkel, K. J., et al. demonstrated that the individually cultured embryos growing at different developmental rates consume pyruvate, lactate, acetate, isoleucine, leucine, valine, threonine, alanine, methionine, lysine, glycine, arginine, phenylalanine, histidine tryptophan, and tyrosine in varying amounts from spent culture medium [55]. For instance, significantly higher levels of valine, leucine, and isoleucine consumption by 16-cell fast growing embryos compared with their slow growing counterparts (developmentally delayed by 12–24 h) were found using the proton nuclear magnetic resonance method. Decreased leucine levels in the embryo culture medium correlated with the pregnancy rate in a study by Brison et al. [56]. These data show that it is important to characterize the growing embryo not only by morphological criteria, but by metabolomic and transcriptomic profiles, as well to assess an embryo’s developmental and implantation potential. 

**Figure 6:**
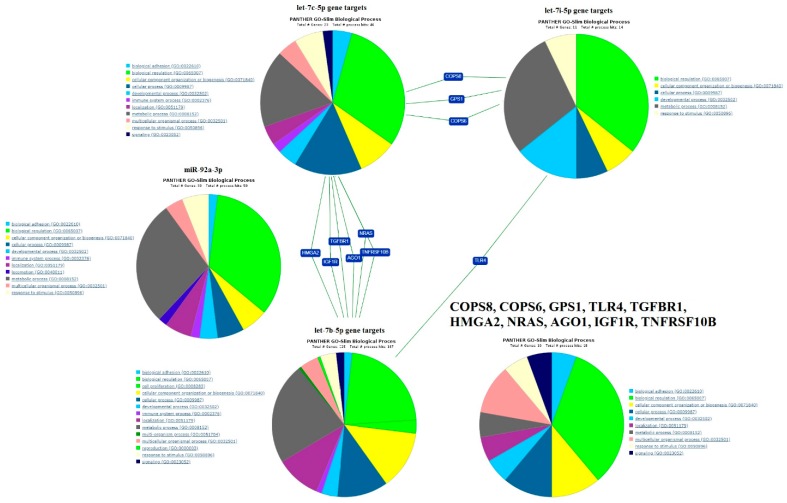
Functional classification of genes targeted by let-7b-5p, let-7c-5p, let-7i-5p, and miR-92a-3p from miRtargetlink and the PANTHER databases.

The miRtargetlink database was used for hsa-let-7c-5p, since no target genes were detected for it by DianaTools_Tarbase. The miRtargetlink contains information on “miRNA/gene target” interactions confirmed by reporter analysis as strong interactions. The list of gene targets of miR-92a-3p, let-7b-5p, let-7c-5p, and let-7i-5p were subjected to ontology and pathway analysis using PANTHER Classification System (Available online: http://pantherdb.org/) and were subsequently classified based on their biological process (Figure 6). Among them, the common target genes of let-7c-5p and let-7i-5p are *GPS1*, *COPS6*, and *COPS8*. *GPS1* is a G Protein Pathway Suppressor 1 known to suppress mitogen-activated signal transduction in mammalian cells. *COPS6* and *COPS8* are subunits 6 and 8 of COP9 Signalosome and are involved in various cellular and developmental processes, for instance, in the regulation of the ubiquitin (Ubl) conjugation pathway. Let-7b-5p and let-7c-5p regulate the expression level of High Mobility Group AT-Hook 2 (*HMGA2*), which is an essential component of the enhancesome and acts as a transcriptional regulating factor; Neuroblastoma RAS proto-oncogene (*NRAS*), which has intrinsic GTPase activity and controls cell proliferation and anti-apoptosis pathways [57]; *AGO1* (Argonaute 1, RISC catalytic component), which degrades and represses the translation of mRNA bound to miRNA as well as performing transcriptional gene silencing of promoter regions bound to short antigene RNAs; *IGF1R* (Insulin Like Growth Factor 1 Receptor, implicated in cell growth and survival control); *TGFBR1* (Transforming Growth Factor Beta Receptor 1, involved in the regulation of cellular processes, including division, differentiation, motility, adhesion, and death); and *TNFRSF10B* (tumor necrosis factor Receptor Superfamily Member 10b, causes cell apoptosis through adapter molecule Fas Associated Via Death Domain (FADD) and effector caspases). The only common experimentally proven target gene of let-7b-5p and let-7i-5p is *TLR4* (Toll Like Receptor 4, transmembrane cell-surface receptor), which was initially discovered in D. Melanogaster as a gene controlling body patterning during embryonic development [58] and plays a key role in the innate immune system [59].

The essential roles of the let-7 and miR-92 family in determining the blastocyst developmental and implantation potential were confirmed by data from Kim J et al. [60]. They showed a significant increase in the expression level of let-7b-5p and miR-92a-3p in outgrowth embryos compared with blastocysts and non-outgrowth embryos. Regulation of the «Mucin type O-Glycan biosynthesis signaling pathway (hsa00512)» under the control of let-7b-5p, let-7c-5p, and let-7i-5p according to the DianaTools database (Table 4) as well as the regulation of the expression level of IGF1R under the influence of let-7b-5p according to the miRtargetlink database (see above) emphasizes the important roles of these miRNA subtypes in implantation processes, since Mucin 1, being an integral transmembrane mucin glycoprotein expressed on the apical surface of the endometrium, acts as an inhibitor of embryo attachment, whereas IGF1R increases on the surfaces of the endometrium during the receptive stage and contributes to adhesive interaction with the embryo [29].

## 3. Materials and Methods

### 3.1. Patients

Forty couples aged between 27 and 40 years with reproductive disorders and IVF indications were enrolled in the study, and 87 samples of spent embryo culture medium (Irvine OneStep) were obtained on day 4 after fertilization during IVF cycles. The morphological characteristics of studied embryos were evaluated on day 5 and were as follows: there were blastocysts of excellent (>3AA, *n* = 32), good (3–6 AB, 3–6 BA, 1–2 AA, *n* = 16), fair (3–6 BB, 3–6 AC, 3–6 CA, 1–2 AB, 1–2 BA, *n* = 11), and poor quality (1–6 BC, 1–6 CB, 1–6 CC, 1–2 BB, *n* = 6) according to the Gardner grading scale [2], as well as morula embryos (*n* = 14) and 3–10 cell embryos (*n* = 8). Out of the 87 embryos, 7 morulas, 3 cavernous morulas, and 38 blastocysts were transferred to the uterus at 5 or 6 days after fertilization. When ovarian hyperstimulation syndrome risk was present, the freeze-all strategy was applied. Before initiation of the IVF program, the following information from a couple was analyzed: medical history, hormonal profile, rhesus blood group system, blood coagulability, infection status, Papanicolaou test, pelvic ultrasound data, and spermogram. There were no statistically significant age or anthropometric measurement differences between patients enrolled in the current study. Patients with normal ovarian reserve, a regular menstrual cycle, and without any extragenital diseases were selected to minimize the influence of maternal factors on embryo implantation. The inclusion criteria were as follows: patients aged 20–37 years old with normal ovarian reserve, tubal factor infertility, and a regular menstrual cycle. The exclusion criteria were patients with contraindications for IVF treatment including extragenital diseases, stage 3–4 moderate and severe endometriosis, polycystic ovary syndrome, endometrial pathology, intramural and subserosal myomas of more than 4 cm, submucosal myomas distorting the uterine cavity, genetic disorders, congenital genitourinary anomalies, a history of invasive surgery on the ovaries, or severe male infertility. 

Forty patients with tubal infertility factor were enrolled in the current study. All patients underwent a short antagonist protocol. Gonadotropin stimulation commenced on day 3–4 of the menstrual cycle. Subcutaneous administration of the gonadotropin-releasing hormone (GnRH) antagonist began when the follicular size was more than or equal to 14 mm. All patients had routine monitoring via transvaginal sonography and hormonal profiling of follicle-stimulating hormone (FSH), luteinizing hormone (LH), estrogen, and progesterone levels. The mature oocytes were retrieved after 34–36 h of human chorionic gonadotropin (HCG) injection. The collected matured oocytes were fertilized in vitro using IVF and the intracytoplasmic sperm injection (ICSI) method. Progesterone vaginal suppository or dydrogesterone enhancesome urred daily after oocyte retrieval and continued until the result of the first blood pregnancy test. All embryos were cultivated in microdrops in the incubator. The assisted hatching procedure was done in individual cases. Single embryo transfer (SET) was performed on day 5 after oocyte retrieval. If the pregnancy test was positive, the patient continued progesterone supplementation until 8 weeks of pregnancy. In the case of a negative result, the medication was discontinued. 

All patients were divided into three groups depending on the IVF program result (Table 5).

Group I: 22 patients who had ovarian stimulation and SET in a stimulated cycle with a negative pregnancy blood test.
Group II: 18 patients who had ovarian stimulation and SET in a stimulated cycle with a positive pregnancy blood test.
Group III: 3 patients from group I who had implantation failure in the previous stimulated cycle and frozen-thawed (FT) embryo transfer with a positive result. All embryos were thawed at the blastocyst stage. The endometrium was prepared with the exogenous administration of oral micronized estradiol forms and progesterone. FT embryo transfer was performed on day 19–20 of the menstrual cycle. The endometrium thickness at the day of embryo transfer was 9–12 mm.

The ethics committee of the National Medical Research Center for Obstetrics, Gynecology, and Perinatology, named after Academician V.I. Kulakov of Ministry of Healthcare of the Russian Federation, approved this study (Ethic’s committee approval protocol No13, approval date: 6 December 2013).

### 3.2. RNA Isolation from Embryo Culture Medium and Blastocoele Fluid

Twenty-five microliters of embryo culture medium or several nanoliters of blastocoele fluid adjusted to 200 μL with 0.9% NaCl were treated with 1000 µL of QIAzol Lysis Reagent (Qiagen, Hilden, Germany), followed by mixing with 200 µL of chloroform, centrifugation for 15 min at 12,000 g (4 °C), collection of 600 µL aqua phase, and RNA isolation using the miRNeasy Serum/Plasma Kit (Qiagen, Hilden, Germany). 

### 3.3. sncRNA Deep Sequencing

cDNA libraries were synthesized using 7 µL of the 14 µL total RNA column eluate (miRNeasy Serum/Plasma Kit, Qiagen, Hilden, Germany), extracted from embryo culture medium and blastocoele fluid using the NEBNext^®^ Multiplex Small RNA Library Prep Set for Illumina^®^ (Set11 and Set2, New England Biolab^®^, Frankfurt am Main, Germany), amplified for 30 PCR cycles, and sequenced on the NextSeq 500 platform (Illumina, San Diego, CA, USA). The adapters were removed with Cutadapt. All trimmed reads shorter than 16 bp and longer than 35 bp were filtered, and only reads with a mean quality higher than 15 were retained. The remaining reads were mapped to the GRCh38.p15 human genomes miRBase v21 and piRNABase with the bowtie aligner [61]. Aligned reads were counted with the featureCount tool from the Subread package [62] and with the fracOverlap 0.9 option, so the whole read was forced to have a 90% intersection with sncRNA features. Differential expression analysis of the sncRNA count data was performed with the DESeq2 package [63].

### 3.4. Reverse Transcription and Quantitative Real-Time PCR

Five microliters of the 14 µL total RNA column eluate (miRNeasy Serum/Plasma Kit, Qiagen, Hilden, Germany) extracted from the embryo culture medium was converted into cDNA in a reaction mixture (20 µL) containing 1x Hispec buffer, 1x Nucleics mix, and miScript RT, according to the miScript^®^ II RT Kit protocol (Qiagen, Hilden, Germany); then, the sample volume was adjusted with deionized water to 200 µL. The synthesized cDNA (3 µL) was used as a template for real-time PCR using a forward primer specific for the studied sncRNA (Table 1) and the miScript SYBR Green PCR Kit (Qiagen, Hilden, Germany). The following PCR conditions were used: (1) 15 min at 95 °C and (2) 40 cycles at 94 °C for 15 s, an optimized annealing temperature (45–60 °C) for 30 s, and 70 °C for 30 s, followed by heating the reaction mixture from 65 to 95 °C by 0.1 °C to plot the melting curve of the PCR product in a StepOnePlus™ thermocycler (Applied Biosystems, Foster City, CA, USA). The relative expression of sncRNA in the embryo culture medium was determined by the ∆∆Ct method using hsa-piR023338 (DQ601914, GenBank, available online: https://www.ncbi.nlm.nih.gov/genbank/) as the reference RNA and culture medium without any embryo incubated for 4 days at 37 °C as a reference sample. hsa-piR023338 was chosen as the reference RNA due to its identical expression level in all 87 analyzed samples.

### 3.5. Data Processing

Scripts written in R were used for the resulting data processing [62,64]. The Shapiro–Wilk test was used to test whether the analyzed parameters were normally distributed. The χ2 test was used for comparing categorical variables. The ANOVA test was used for the analysis of the three groups of normally distributed parameters. Finally, the Mann–Whitney U-test was used for the pairwise comparison of the non-normally distributed parameters. Absolute numbers (N) and percentages of the total number of patients in a group (P) in the N (P%) format were used to describe categorical binary data. The arithmetic mean (M) and standard deviation (SD) in M (SD) format were used to evaluate the normally distributed quantitative data. Non-normally distributed parameters were described as medians (Me) and quartiles (Q1 and Q3) in the Me (Q1; Q3) format. The Spearman correlation analysis was performed, since both quantitative and qualitative data were analyzed. A 95% confidence interval for the correlation coefficients was determined using the Fisher transform strategy. The threshold for the statistical significance was *p* ≤ 0.05. The *p*-value was specified in the *p* < 0.001 format if it was less than 0.001. In addition, the morphological characteristics of the embryos and the obtained experimental data were analyzed using PLS-DA [65].

## 4. Conclusions

In recent years, the attention of scientists has been drawn to the study of the role of sncRNA in embryogenesis. In the course of the present research work, a complex interrelation of sncRNA expression patterns in the culture media of embryos at various development grades on day 4 after fertilization was revealed. These findings probably reflect the manifestation of the fine regulation system of signaling pathways which is necessary for implementation of the embryogenesis program. The pathway analysis of miRNA and piRNA target genes emphasizes the role of sncRNA described in this article in the control of chromatin structure, genome stability, DNA replication, gene transcription, protein synthesis, cell survival, migration and invasion, cell cycle progression, and cellular proliferation and differentiation, i.e., the processes that determine the normal course of gametogenesis and embryogenesis. Correlations between the sncRNA expression patterns and the number of oocyte–cumulus complexes, metaphase II oocytes, and two pronuclei embryos as well as the spermatozoid count, the percentage of linearly motile spermatozoids, the embryo development grade, and embryo implantation provide evidence of the roles of these molecules in human reproductive system regulation.

## Figures and Tables

**Table 1:** Small non-coding RNA (sncRNA) identification by next generation sequencing (NGS) and real time polymerase chain reaction (PCR) (if Ct > 35 cycles in most of the studied samples, these data were excluded from the analysis, “+”—data suitable for analysis).

sncRNA	NGS Read Count (Blastocoele Liquid/Embryo Culture Medium)	5′–3′ Sequence of Sense Primer, Tm (Melting Temperature)	PCR (Embryo Culture Medium)
hsa-let-7b-5p	527/282	Hs_let-7b_1 miScript Primer Assay, Cat.No. MS00003122, Tm = 55 °C	+
hsa-let-7a-5p	537/258	Hs_let-7a_2 miScript Primer Assay, Cat.No. MS00031220, Tm = 55 °C	Ct > 35
hsa-miR-99a-5p	351/145	Hs_miR-99a_2 miScript Primer Assay, Cat.No. MS00032158, Tm = 55 °C	Ct > 35
hsa-miR-148a-3p	194/136	Hs_miR-148a_1 miScript Primer Assay, Cat.No. MS00003556, Tm = 55 °C	Ct > 35
hsa-let-7i-5p	239/114	Hs_let-7i_1 miScript Primer Assay, Cat.No. MS00003157, Tm = 55 °C	+
hsa-miR-26a-5p	209/111	Hs_miR-26a_2 miScript Primer Assay, Cat.No. MS00029239, Tm = 55 °C	Ct > 35
hsa-let-7c-5p	192/110	Hs_let-7c_1 miScript Primer Assay, Cat.No. MS00003129, Tm = 55 °C	+
hsa-let-7g-5p	145/89	Hs_let-7g_2 miScript Primer Assay, Cat.No. MS00008337, Tm = 55 °C	Ct > 35
hsa-miR-92a-3p	144/91	Hs_miR-92_1 miScript Primer Assay, Cat.No. MS00006594, Tm = 55 °C	+
hsa-miR-143-3p	135/81	Hs_miR-143_1 miScript Primer Assay, Cat.No. MS00003514, Tm = 55 °C	+
hsa-miR-125b-5p	69/57	Hs_miR-125b_1 miScript Primer Assay, Cat.No. MS00006629, Tm = 55 °C	Ct > 35
hsa-miR-100-5p	77/50	Hs_miR-100_2 miScript Primer Assay, Cat.No. MS00031234, Tm = 55 °C	Ct > 35
hsa-miR-125a-5p	63/36	Hs_miR-125a_1 miScript Primer Assay, Cat.No. MS00003423, Tm = 55 °C	Ct > 35
hsa-miR-320a	57/30	Hs_miR-320a_1 miScript Primer Assay, Cat.No. MS00014707, Tm = 55 °C	Ct > 35
hsa-miR-27b-3p	56/28	Hs_miR-27b_2 miScript Primer Assay, Cat.No. MS00031668, Tm = 55 °C	Ct > 35
hsa-miR-200c-3p	47/28	Hs_miR-200c_1 miScript Primer Assay, Cat.No. MS00003752, Tm = 55 °C	Ct > 35
Hsa-piR020401, DQ598029	0/23,808	GGCTGGTCTCGAACTCCTGACCTCAGGT, Tm = 45 °C	+
Hsa-piR023338, DQ601914	0/27,727	TAGTCCCAGCTACTTGGGAGGCTGAGGCA, Tm = 45 °C	+
Hsa-piR019675, DQ596992	2708/2645	GCAATAACAGGTCTGTGATGCCCTTAGA, Tm = 53 °C	+
Hsa-piR016735, DQ593039	1676/1240	CCTGGGAATACCGGGTGCTGTAGGCTTA, Tm = 50 °C	+
Hsa-piR017716, DQ594453	620/680	TTCCCTGGTGGTCTAGTGGTTAGGATTCGGC, Tm = 45 °C	+
Hsa-piR020326, DQ597916	1810/2540	GGCATTGGTGGTTCAGTGGTAGAATTCTCGC, Tm = 60 °C	+
Hsa-piR020497, DQ598177	291/350	TGTAGCTCAGTGGTAGAGCGCGTGCT, Tm = 45 °C	+
Hsa-piR022296, DQ600515	0/10,609	TACTCAGGAGGCTGAGGCAGGAGAATGGC, Tm = 45 °C	+

**Table 2:** Pairwise comparison of the fold changes in the expression level of sncRNAs in the sample groups shown in Figure 3.

sncRNA	Group 1–Group 2	*p*-Value	Group 1, Me(Q1, Q3) *	Group 2, Me(Q1, Q3) *
let-7b-5p	“3–10 cell embryon”–“morula”	0.04	6.16 (4.08, 7.86)	10.1 (6.42, 12.86)
	“morula”–“fair quality blastocyst”	0.02	10.1 (6.42, 12.86)	4.18 (1.94, 6.71)
	“morula”–“excellent quality blastocyst”	0.05	10.1 (6.42, 12.86)	7.24 (3.48, 10.07)
let-7i-5p	“morula”–“poor quality blastocyst”	0.02	2.2 (0.87, 4.33)	6.2 (5.19, 6.34)
	“cavernous morula”–“poor quality blastocyst”	0.01	3.17 (2.67, 3.89)	6.2 (5.19, 6.34)
	“poor quality blastocyst”–“excellent quality blastocyst”	0.01	6.2 (5.19, 6.34)	3.26 (2.18, 4.52)
piR020401	“morula”–“fair quality blastocyst”	0.02	2.12 (1.94, 2.2)	1.92 (1.43, 2.01)
	“fair quality blastocyst”–“good quality blastocyst”	<0.001	1.92 (1.43, 2.01)	2.14 (2.05, 2.27)
piR16735	“3–10 cell embryon”–“cavernous morula”	<0.001	1.21 (1.21, 1.88)	6.78 (3.97, 14.92)
	“3–10 cell embryon”–“good quality blastocyst”	0.01	1.21 (1.21, 1.88)	6.3 (3.51, 8.2)
	“cavernous morula”–“fair quality blastocyst”	0.04	6.78 (3.97, 14.92)	1.74 (1.41, 3.85)
	“cavernous morula”–“excellent quality blastocyst”	0.02	6.78 (3.97, 14.92)	2.61 (1.62, 5.62)
piR17716	“poor quality blastocyst”–“good quality blastocyst”	0.04	0.72 (0.69, 0.73)	0.88 (0.73, 0.98)
piR19675	“3–10 cell embryo”–“good quality blastocyst”	0.02	0.1 (0.05, 0.36)	1.26 (1.08, 1.63)
	“3–10 cell embryo”–“excellent quality blastocyst”	0.03	0.1 (0.05, 0.36)	1.23 (0.7, 1.5)
piR20326	“3–10 cell embryo”–“good quality blastocyst”	0.02	0.06 (0.04, 0.06)	2.88 (1.09, 3.92)

* The data are presented as medians (Me) and quartiles Q1 and Q3 in the format Me (Q1; Q3).

**Table 3:** Genomic localization and potential piwiRNA target genes.

Genbank ID	PiRBase ID for piRNAs	Location in hg38 *	RefSeq Gene	Repeat Information	Potential Gene Targets
hsa_piR_017716|gb|DQ594453|Homo	piR-hsa-24672, aliases piR-60565, PIR55564	chr6_GL000255v2_alt:247775-247806: +		Name: tRNA-Glu-GAG, Family: tRNA, Class: tRNA	
hsa_piR_020401|gb|DQ598029|Homo	piR-hsa-28244, aliases piR-36095 PIR59140	chr6_GL000253v2_alt:2984874-2984902: −	LY6G5C NM_025262	Name: AluSx, Family: Alu, Class: SINE	*PAK3,CXorf38,FRRS1L,C9orf85,EEF1D,CACNA2D1,RBM28,POLH,TRIM52,FER,NAF1,NSUN7,LIAS,PHC3,RABL2B,KIAA1755,ZNF13,PHF20,ZHX3,TCFL5,CARF*
hsa_piR_016735|gb|DQ593039|Homo	piR-hsa-23317, aliases piR-33151 PIR54150	chr6:4428052-4428083: +		Name: 5S, Family: rRNA, Class: rRNA	
hsa_piR_019675|gb|DQ596992|Homo	piR-hsa-27282, aliases piR-35058 PIR58103	chr14:89875053-89875081: −	EFCAB11 NM_001284269;NM_145231; NM_001284267	Name: SSU-rRNA_Hsa, Family: rRNA, Class: rRNA	*MDN1,MLKL,METTL22, ARHGEF10L*
		chr21:8211110-8211138: +	RNA18SN2 NR_146146; RNA18SN4 NR_146119; RNA18SN3 NR_146152; RNA18SN5 NR_003286; RNA45SN2 NR_146144; LOC100507412 NR_038958; RNA18SN1 NR_145820	Name: SSU-rRNA_Hsa, Family: rRNA, Class: rRNA	
		chr21:8255319-8255347: +	RNA28SN5 NR_003287	Name: SSU-rRNA_Hsa, Family: rRNA, Class: rRNA	
		chr21:8394145-8394173: +	LOC100507412 NR_038958; RNA28SN5 NR_003287; RNA18SN2 NR_146146; RNA18SN5 NR_003286; RNA45SN3 NR_146151; RNA18SN3 NR_146152; RNA18SN4 NR_146119; RNA18SN1 NR_145820;	Name: SSU-rRNA_Hsa, Family: rRNA, Class: rRNA	
		chr21:8438355-8438383: +	RNA45SN1 NR_145819; RNA18SN2 NR_146146; RNA45SN5 NR_046235; RNA18SN5 NR_003286; RNA18SN3 NR_146152; RNA18SN4 NR_146119; RNA18SN1 NR_145820;	Name: SSU-rRNA_Hsa, Family: rRNA, Class: rRNA	
		chr22_KI270733v1_random:127410-127438: +	RNA45SN1 NR_145819; RNA45SN5 NR_046235; RNA45SN2 NR_146144; RNA45SN4 NR_146117; RNA18SN5 NR_003286; RNA18SN3 NR_146152; RNA18SN4 NR_146119; RNA18SN2 NR_146146; RNA18SN1 NR_145820;	Name: SSU-rRNA_Hsa, Family: rRNA, Class: rRNA	
		chr22_KI270733v1_random:172491-172519: +	RNA18SN3 NR_146152; RNA18SN5 NR_003286; RNA18SN4 NR_146119; RNA18SN2 NR_146146; RNA18SN1 NR_145820;	Name: SSU-rRNA_Hsa, Family: rRNA, Class: rRNA	
hsa_piR_020497|gb|DQ598177|Homo	piR-hsa-28392, aliases piR-36243 PIR59288	chr6:28863725-28863757: −, chr6_GL000250v2_alt:129309-129341: −, chr6_GL000251v2_alt:354265-354297: −, chr6_GL000252v2_alt:129327-129359: −, chr6_GL000253v2_alt:129283-129315: −, chr6_GL000254v2_alt:129319-129351: −, chr6_GL000255v2_alt:129307-129339: −, chr6_GL000256v2_alt:172970-173002: −		Name: tRNA-Ala-GCY, Family: tRNA, Class: tRNA	
hsa_piR_020326|gb|DQ597916|Homo	piR-hsa-28131, aliases piR-35982 PIR59027	chr1:16545979-16546010: −, chr1:16861919-16861950: +		Name: tRNA-Gly-GGG, Family: tRNA, Class: tRNA	
		chr16:70789505-70789536: +	VAC14 NM_001351157, NM_018052	Name: tRNA-Gly-GGY, Family: tRNA, Class: tRNA	
		chr3:15507990-15508021: +	COLQ NM_080539, NM_005677	Name: tRNA-Gly-GGG, Family: tRNA, Class: tRNA	
		chr6:27902948-27902979: −		Name: tRNA-Gly-GGY, Family: tRNA, Class: tRNA	
hsa_piR_022296|gb|DQ600515|Homo	piR-hsa-30715, aliases piR-38581 PIR61626	chr18:13769187-13769216: +		Name: AluY, Family: Alu, Class: SINE	*NR6A1,RELL1,IL17RB,POLA2,PLPP4,PRPF3,C3orf70,CDH18,ZNF726,UGCG,IL17RD,MCF2L2,TBC1D24,AMN1,KIN,PLPP4,ZNF124,ACTR8,LPP,CPT1A,ZNF320*
hsa_piR_023338|gb|DQ601914|Homo	piR-hsa-30937, aliases piR-39980 PIR63025	chr17:42974237-42974266: +	PTGES3L NM_001142654, NM_001142653, NM_001261430; PTGES3L-AARSD1 NM_025267, NM_001136042;	Name: AluSq, Family: Alu, Class: SINE	

* ”+” denotes sense strand which contains the exact nucleotide sequence to the messenger RNA (Mrna); “−“ denotes antisense strand, serves as the template for the transcription and contains complementary nucleotide sequence to the transcribed mRNA.

**Table 4:** Predicted and experimentally supported miRNA targets.

**DianaTools microT-CDS Algorithm for the Prediction of miRNA Targets in Both the three prime untranslated regions (3’UTRs) and Coding Sequence (CDS)**			
**Kyoto Encyclopedia of Genes and Genomes (KEGG) Pathway**	***p*-Value**	**Number of Genes**	**miRNAs**
ECM–receptor interaction	< 1 × 10^−325^	15	hsa-let-7b-5p, hsa-let-7c-5p, hsa-let-7i-5p, hsa-miR-92a-3p
Mucin type O-Glycan biosynthesis	4.21 × 10^−11^	5	hsa-let-7b-5p, hsa-let-7c-5p, hsa-let-7i-5p
Glycosaminoglycan biosynthesis—chondroitin sulfate/dermatan sulfate	8.44 × 10^−8^	4	hsa-let-7b-5p, hsa-let-7c-5p, hsa-let-7i-5p
Amoebiasis	2.48 × 10^−7^	11	hsa-let-7b-5p, hsa-let-7c-5p, hsa-let-7i-5p
Signaling pathways regulating stem cell pluripotency	1.19 × 10^−5^	16	hsa-let-7b-5p, hsa-let-7c-5p, hsa-let-7i-5p
Protein digestion and absorption	0.006789	11	hsa-let-7b-5p, hsa-let-7c-5p, hsa-let-7i-5p
PI3K-Akt signaling pathway	0.009286	27	hsa-let-7b-5p, hsa-let-7c-5p
Wnt signaling pathway	0.017555	11	hsa-let-7c-5p
**DianaTools_TargetScan—Predicts Biological Targets of miRNAs Considering Matches to 3’ UTRs**			
Mucin type O-Glycan biosynthesis (hsa00512)	4.43 × 10^−12^	1	hsa-let-7b-5p, hsa-let-7c-5p, hsa-let-7i-5p
Valine, leucine, and isoleucine biosynthesis (hsa00290)	1.79 × 10^−8^	1	hsa-let-7b-5p, hsa-let-7c-5p, hsa-let-7i-5p
Signaling pathways regulating stem cell pluripotency (hsa04550)	7.77 × 10^−6^	6	hsa-let-7b-5p, hsa-let-7c-5p, hsa-let-7i-5p
Biosynthesis of amino acids (hsa01230)	0.000132	2	hsa-let-7b-5p, hsa-let-7c-5p, hsa-let-7i-5p
2-Oxocarboxylic acid metabolism (hsa01210)	0.000166	1	hsa-let-7b-5p, hsa-let-7c-5p, hsa-let-7i-5p
Oocyte meiosis (hsa04114)	0.008317	3	hsa-let-7b-5p, hsa-let-7c-5p
Valine, leucine, and isoleucine degradation (hsa00280)	0.026219	1	hsa-let-7c-5p
Arginine and proline metabolism (hsa00330)	0.026219	2	hsa-let-7c-5p
**DianaTools_Tarbase—Database of Experimentally Supported miRNA Targets**			
Lysine degradation (hsa00310)	6.06959 × 10^−13^	20	hsa-let-7b-5p, hsa-let-7i-5p, hsa-miR-92a-3p
Cell cycle (hsa04110)	1.13021 × 10^−11^	52	hsa-let-7b-5p, hsa-let-7i-5p, hsa-miR-92a-3p
Viral carcinogenesis (hsa05203)	5.62537 × 10^−10^	60	hsa-let-7b-5p, hsa-let-7i-5p, hsa-miR-92a-3p
Hepatitis B (hsa05161)	5.35744 × 10^−08^	54	hsa-let-7b-5p, hsa-let-7i-5p, hsa-miR-92a-3p
Oocyte meiosis (hsa04114)	1.75682 × 10^−07^	41	hsa-let-7b-5p, hsa-let-7i-5p, hsa-miR-92a-3p
Chronic myeloid leukemia (hsa05220)	2.5576 × 10^−7^	27	hsa-let-7b-5p, hsa-let-7i-5p, hsa-miR-92a-3p
Hippo signaling pathway (hsa04390)	4.05654 × 10^−7^	50	hsa-let-7b-5p, hsa-let-7i-5p, hsa-miR-92a-3p
Proteoglycans in cancer (hsa05205)	1.70389 × 10^−6^	64	hsa-let-7b-5p, hsa-let-7i-5p, hsa-miR-92a-3p
Thyroid hormone signaling pathway (hsa04919)	2.7327 × 10^−6^	39	hsa-let-7b-5p, hsa-let-7i-5p, hsa-miR-92a-3p
Adherens junctions (hsa04520)	3.42243 × 10^−6^	30	hsa-let-7i-5p, hsa-miR-92a-3p
FoxO signaling pathway (hsa04068)	2.15597 × 10^−5^	50	hsa-let-7b-5p, hsa-let-7i-5p, hsa-miR-92a-3p
ECM–receptor interaction (hsa04512)	2.47672 × 10^−5^	12	hsa-let-7i-5p
Bacterial invasion of epithelial cells (hsa05100)	4.61176 × 10^−5^	28	hsa-let-7b-5p, hsa-let-7i-5p, hsa-miR-92a-3p
Glioma (hsa05214)	7.08143 × 10^−5^	20	hsa-let-7b-5p, hsa-let-7i-5p
Pathways in cancer (hsa05200)	0.000154251	99	hsa-let-7i-5p, hsa-miR-92a-3p
Colorectal cancer (hsa05210)	0.000634129	25	hsa-let-7b-5p, hsa-let-7i-5p, hsa-miR-92a-3p
Thyroid cancer (hsa05216)	0.000693712	12	hsa-let-7b-5p, hsa-let-7i-5p, hsa-miR-92a-3p
Prostate cancer (hsa05215)	0.001016403	35	hsa-let-7b-5p, hsa-let-7i-5p, hsa-miR-92a-3p
Epstein–Barr virus infection (hsa05169)	0.002168481	51	hsa-let-7b-5p, hsa-let-7i-5p
MAPK signaling pathway (hsa04010)	0.003378721	59	hsa-let-7b-5p, hsa-let-7i-5p, hsa-miR-92a-3p
Endocytosis (hsa04144)	0.003828609	62	hsa-let-7b-5p, hsa-let-7i-5p, hsa-miR-92a-3p
Huntington’s disease (hsa05016)	0.003922408	50	hsa-let-7b-5p, hsa-miR-92a-3p
Small cell lung cancer (hsa05222)	0.004508148	24	hsa-let-7b-5p, hsa-let-7i-5p
TGF-beta signaling pathway (hsa04350)	0.006182085	29	hsa-let-7b-5p, hsa-let-7i-5p, hsa-miR-92a-3p
p53 signaling pathway (hsa04115)	0.007856274	20	hsa-let-7b-5p, hsa-let-7i-5p
Melanoma (hsa05218)	0.008148048	18	hsa-let-7b-5p, hsa-let-7i-5p
Shigellosis (hsa05131)	0.009612655	13	hsa-miR-92a-3p
Bladder cancer (hsa05219)	0.009747186	14	hsa-let-7b-5p, hsa-let-7i-5p
Signaling pathways regulating pluripotency of stem cells (hsa04550)	0.01296196	26	hsa-miR-92a-3p
Estrogen signaling pathway (hsa04915)	0.01345837	17	hsa-let-7i-5p
Transcriptional misregulation in cancer (hsa05202)	0.01729337	45	hsa-let-7i-5p, hsa-miR-92a-3p
Ubiquitin mediated proteolysis (hsa04120)	0.01776272	34	hsa-let-7b-5p
Protein processing in endoplasmic reticulum (hsa04141)	0.02539808	38	hsa-let-7b-5p, hsa-let-7i-5p
RNA transport (hsa03013)	0.02748059	41	hsa-let-7b-5p
Valine, leucine and isoleucine biosynthesis (hsa00290)	0.03746544	2	hsa-miR-92a-3p

**Table 5:** Comparison of the clinical and demographic characteristics of patients enrolled in the current study.

Clinical and Demographic Characteristics	Group I (Implantation Failure)—25 Patients	Group II (Successful Implantation)—14 Patients	Group III (Successful Implantation in A Frozen Embryo Transfer Protocol)—4 Patients	*p*
Body mass index (BMI) *	22.1 (2.0)	22.2 (1.4)	22.0 (1.8)	>0.05
Menstrual cycle length (days) *	29.9 (1.6)	28.4 (0.9)	29.0 (2.7)	>0.05
Average age (years) *	32.3 (3.5)	32.0 (3.1)	30.0 (2.4)	>0,05
Follicle-stimulating hormone level on day 2–3 of menstrual cycle (mIU/mL) *	7.8 (1.4)	7.2 (1.4)	5.8 (0.5)	>0.05
Anti-mullerian hormone level (ng/mL) *	2.4 (1.0)	2.4 (0.7)	2.8 (0.5)	>0.05
Antral follicle count on day 2–3 of the menstrual cycle *	8.2 (1.6)	7.8 (1.5)	8.3 (1.0)	>0.05
Male factor infertility **	18 patients (64%) had male factor infertility	14 patients (78%) had male factor infertility	2 patients (50%) had male factor infertility	>0.05
Primary infertility **	13 patients (46%) had primary infertility	8 patients (44%) had primary infertility	2 patients (50%) had primary infertility	>0.05
Assisted hatching **	In 20 (71%) patients, assisted hatching was performed	In 10 (56%) patients, assisted hatching was performed	In 2 (50%) patients, assisted hatching was performed	>0.05
Number of oocytes retrieved *	7.8 (3.7)	7.4 (4.5)	8.0 (3.7)	>0.05
Number of blastocysts *	1.4 (1.3)	1.4 (1.2)	2.8 (2.0)	>0.05

* Data are presented as an arithmetic mean (M) and standard deviation (SD) in the format of M(SD), specifying the significant differences when using ANOVA test; ** data are presented as absolute N numbers and percentages of the total number of patients in group P in the format of N(P%), specifying the significant differences when using the χ2 test. Comment: Group III (successful implantation in a frozen embryo transfer protocol) coincides with Group I (implantation failure), since patients from Group I were transferred to Group III after their previous failed IVF.
